# Dynamic probabilistic threshold networks to infer signaling pathways from time-course perturbation data

**DOI:** 10.1186/1471-2105-15-250

**Published:** 2014-07-22

**Authors:** Narsis A Kiani, Lars Kaderali

**Affiliations:** Technische Universität Dresden, Medical Faculty Carl Gustav Carus, Institute for Medical Informatics and Biometry, Fetscherstr. 74, 01307 Dresden, Germany

## Abstract

**Background:**

Network inference deals with the reconstruction of molecular networks from experimental data. Given *N* molecular species, the challenge is to find the underlying network. Due to data limitations, this typically is an ill-posed problem, and requires the integration of prior biological knowledge or strong regularization. We here focus on the situation when time-resolved measurements of a system’s response after systematic perturbations are available.

**Results:**

We present a novel method to infer signaling networks from time-course perturbation data. We utilize dynamic Bayesian networks with probabilistic Boolean threshold functions to describe protein activation. The model posterior distribution is analyzed using evolutionary MCMC sampling and subsequent clustering, resulting in probability distributions over alternative networks. We evaluate our method on simulated data, and study its performance with respect to data set size and levels of noise. We then use our method to study EGF-mediated signaling in the ERBB pathway.

**Conclusions:**

Dynamic Probabilistic Threshold Networks is a new method to infer signaling networks from time-series perturbation data. It exploits the dynamic response of a system after external perturbation for network reconstruction. On simulated data, we show that the approach outperforms current state of the art methods. On the ERBB data, our approach recovers a significant fraction of the known interactions, and predicts novel mechanisms in the ERBB pathway.

**Electronic supplementary material:**

The online version of this article (doi:10.1186/1471-2105-15-250) contains supplementary material, which is available to authorized users.

## Background

The availability of high throughput experimental platforms has transformed molecular biology into a data-rich science. However, the development of computational and mathematical tools to extract and interpret the wealth of information hidden in these data is lagging behind. For example, genome wide RNA interference screens have enabled the phenotypic characterization of genes at an unprecedented scale in living cells [1]. However, the interpretation of such data and the placement of hits in their functional and temporal context in cellular pathways remains a major challenge [2, 3]. Machine learning can address many of the questions arising from the interpretation of large scale molecular biological data, and has become an important tool of bioinformatics and systems biology. Automatic network inference deals with the reconstruction of regulatory or signaling networks directly from experimental data using statistical or machine learning approaches, a field that has received significant attention in the last decade. There is a wide variety of different approaches available that can be used to infer genetic regulatory or signal transduction networks from experimental data. Approaches employed include Bayesian networks [4–13] and dynamic Bayesian networks [14–18], Boolean models [19–21], auto-regressive models, correlation-based and mutual-information based models, clustering techniques, differential equation models, and others [22–27]. These methods differ in the level of detail at which they reconstruct networks, and in their underlying assumptions and data requirements. Some produce an undirected graph, where edges do not indicate which gene or protein in a connected pair is the activator, and which is the activated gene or protein [23]. Others specify the regulator with directed edges [14], and a few label the edges with kinetic parameters [28].

There are a number of successful applications of network inference approaches to elucidate cellular signaling pathways, including meta-approaches integrating different methods [29]. Among the successful applications, for example, Sachs *et al.* used Bayesian networks to reconstruct cellular protein signaling networks from protein phosphorylation measurements [5]. Nelander *et al.* used a model based on nonlinear differential equations to infer signaling networks in cancer from combinatorial drug perturbation data [30]. Eduati *et al.* demonstrate the integration of literature-knowledge into data driven approaches, and show a successful application to a signaling network related to growth-signaling and inflammation [31]. Ciaccio *et al.* used Bayesian networks and two different mutual-information based approaches to infer signaling networks downstream of the EGF receptor [32]. Hill *et al.* use dynamic Bayesian networks to study signaling in a cancer cell line [18]. Other approaches include nested effects models (NEM) [33], deterministic effects propagation networks (DEPN) [34] or probabilistic Boolean threshold networks (PBTN) [35], and have been applied, for example, to reconstruct signaling networks in the ERBB pathway, or in the innate immune response to infection.

Network reconstruction can be performed from observational data alone. However, the quality of the reconstruction increases substantially if experimental perturbations followed by observations of the system’s dynamic response are available [36]. Surprisingly, while there are many approaches available for time course data, and several approaches for perturbation data, there are only few methods available that can handle both time course data and perturbations at the same time. Exceptions are, for example, the differential equation approach presented by Nelander *et al.* [30], Dynamic Nested Effects Models (DNEM) [37, 38] or Dynamic Deterministic Effects Propagation Models (D-DEPN) [39]. Among the stochastic approaches, DNEMs rely on high-dimensional, indirect readouts of rather qualitative knockdown "effects", such as microarrays performed at different time points after every knockdown, or multidimensional features derived from live cell imaging. Such data are often not available, and also provide only indirect information about the underlying signaling pathway. Fröhlich *et al.* proposed Deterministic Effects Propagation Networks (DEPN) to reconstruct networks from perturbation data with direct observation of involved proteins, measured e.g. using reverse phase protein arrays [34]. However, DEPNs treat each time point as an independent measurement and do not model the time dependent behavior of the system explicitly. Bender *et al.* proposed *Dynamic* DEPNs (D-DEPNs), to take the availability of longitudinal data as well as inhibitory interactions in a network into account [39]. D-DEPNs use the Viterbi algorithm to identify the state transitions of a hidden Markov model from the time course data, with states corresponding to combinations of activities of nodes in the network. A likelihood function is defined to score network models given the estimated state transitions, and the network space is then searched using a genetic algorithm. Due to this underlying procedure, D-DEPNs require relatively long time series (10 time points in the original publication of the method), and have substantial running time requirements if large numbers of different perturbations involving most or all of the proteins in a network are performed.

We here focus on the problem of reconstructing signaling networks from *short* time series with only two or three time points, after a *large* number of different, possibly combinatorial perturbations, targeting most or all of the genes in the network. Such data arise, for example, if RNAi experiments are combined with protein array measurements at few time points after the perturbation, or in time-resolved mass spectrometric assays under different conditions. We propose a new method for the identification of kinetic models of signaling networks from such data, dynamic probabilistic Boolean threshold networks (D-PBTN), which can treat multiple, combinatorial interventions as well as incomplete observations. Through the use of a fairly simple, discrete-state model where proteins are either active or inactive, our method is applicable to qualitative data and does not require detailed quantitative measurements. Furthermore, through the Bayesian framework employed, we can easily handle noisy or missing data, and using MCMC sampling, can analyze full posterior distributions over model parameters, yielding information about different, alternative network topologies that are consistent with the experimental data. Our work builds upon probabilistic Boolean threshold networks (PBTN) regarding the underlying dynamic network model [35], but extends the method in the following aspects: (1) We fully exploit the information from time course data, by explicitely integrating time into the model likelihood. In contrast, PBTNs in their original implementation only exploit observational data taken at a single time point, usually at steady state. (2) PBTNs are limited to relatively small networks of around 7 nodes. Through a more efficient sampling method, the approach presented here can be used for larger networks, effectively more than doubling the feasible network size. (3) PBTNs were implemented with a focus on data with only single downstream observations at a single time point, and therefore similarly to other (non-dynamic) Bayesian network based approaches, do not allow feedback loops in the network. The approach presented here can handle feedback loops in the network. (4) D-PBTNs can handle overexpression and knockdown data, in contrast to PBTNs, which are limited to knockdown data.

Our approach uses discrete states of the proteins in the network, which can be either "on" or "off". In terms of the biological interpretation, this could be phosphorylation networks, in which case edges would correspond to phosphorylation or dephosphorylation events. Alternatively, assuming that proteins are "present" or "absent", edges in the model could also be interpreted as transcriptional regulation. Correspondingly, nodes can represent "activated" protein levels or total protein abundance depending on context.

We evaluate the performance of our method on simulated data, and study its behavior on networks of different sizes, with different amounts of available data and different levels of noise, and compare results against PBTN [35], DEPN [34] and BDAGL, a dynamic Bayesian network based approach [40, 41]. Dynamic DEPNs could not be used for the comparison, as they were unable to handle the short time courses and large number of perturbations. We show inference results for ERBB signaling in breast cancer using DEPN, PBTN and D-PBTN, demonstrating superior performance of D-PBTN, and showing cross-talk between different branches of the ERBB pathway.

## Methods

### Mathematical model

We model a signaling network by a weighted, directed graph . Cycles are permitted. The node set  corresponds to proteins; an edge  from node *v*_*i*_ to *v*_*j*_ represents a regulation of *v*_*j*_ by *v*_*i*_, such as activation or deactivation via (de)phosphorylation. The strength and type of an interaction is specified by , with *w*_*i*,*j*_ > 0 for activations, *w*_*i*,*j*_ < 0 for inactivations and *w*_*i*,*j*_ := 0 if . Given *N*, the number of nodes in the network, we can thus describe the network topology by its *N* × *N* weighted adjacency matrix . We furthermore consider each node *v*_*i*_ a Boolean variable. Model time is assumed discrete, and all nodes are updated simultaneously between two time steps. The state of node *v*_*i*_(*t* + 1) at model time *t* + 1 is described by a stochastic function of the states of all nodes  at model time *t*:
1

The relationship between a target protein and its regulators is hence modeled by a sigmoid function, an ansatz for nonlinear systems with saturation phenomena. Equation (1) activates or deactivates a target protein depending on the weighted sum of incoming regulations. The level of stochasticity in this process is defined by the positive parameter *γ*, whereas *w*_0,*i*_ defines a basal activation probability of *v*_*i*_ in the absence of any regulatory effects.

In case of interventions, the state of a node *v* targeted by perturbation is no longer subject to the stochastic dynamics of the system, instead, its value is fixed through the intervention. We write , *k* ∈ {1,…,*K*}, for the set of nodes targeted simultaneously in perturbation experiment *k*, and assume that a knockdown of  amounts to fixing all nodes  to the "off" state at all model time points after the knockdown. Similarly, overexpression experiments can be simulated by fixing the affected nodes *v*_*i*_ to "on".

Let us now assume that we have observed experimentally the states of the nodes *v*_*i*_ at T different (real) time points , . We note in passing that this normally requires a discretization step, as experimental data are typically measured continuously – see the section on the ERBB data set below. Let us for now assume that the set of experimental observations  consists of measurements of *N* discrete protein states at *T* time points after *K* different perturbations. We write  for the observed activity of node *v*_*i*_ at the real time point  after knockdown of node set . To map real time  to model time *t*, we introduce a parameter vector  to denote the number of model time steps that correspond to the real time elapsed between the different experimental time points , that is,  denotes the number of model time steps according to equation (1) required to transition from real time point  to time point .

Assuming a one-to-one correspondence between model time and real time, that is, assuming that  for all , we can define a likelihood by calculating the product of  according to equation (1) over all nodes *v*, time points *t* and perturbations *k*:
2

If *τ*_*t*_ > 1, one or more intermediate model time steps must be made to transition from experimental time point  to experimental time point . As no experimental observations are available for the intermediate time steps, we must marginalize over unobserved time steps in the evaluation of the likelihood (2). Writing  for the full sequence of model steps, where the sequence of intermediate steps  are unobserved, equation (2) becomes
3

where  comprises the product over all individual nodes as in equation (1), and where the sum is over all possible combinations of values for the intermediate model steps. Missing values in the experimental data can be treated similarly – the likelihood can then be computed by integrating equation (3) over all possible combinations of values for unobserved .

We note that PBTNs are based on a closely related likelihood function, and employ the same underlying model for the relation between nodes, given in equation (1). However, PBTNs differ in a key aspect from the framework presented here: PBTNs do not allow multiple time points in their likelihood, but rather assume that measurements are made at a single time point after knockdown, usually taken at steady state.

### Inclusion of prior knowledge

In many biological settings, available data are insufficient to unambiguously reconstruct the underlying network. In such situations, strict regularization of the objective function using for example maximum parsimony [28], or the inclusion of additional prior biological knowledge [42] can help. Both can be done via Bayes’ theorem using prior distributions on the model parameters Ω. The model posterior is
4

where  is the likelihood function as defined above, *p*(Ω) is an adequately defined prior distribution, and  is a constant normalizing factor that does not depend on Ω and can be neglected in maximization or sampling. The prior can be written as , assuming that the different types of model parameters are independent. Then,  describes our belief about the correct topology, prior to seeing any data, and *p*(*τ*) describes our belief about the speed of signal transduction in the network. Assuming independence of parameters, the full prior can be written as:
5

In the network inference setting, it is unlikely that the true underlying biological network is densely connected. We rather expect a sparse network, where most of the nodes have only few other nodes acting on them [43–47]. This can be mathematically encoded in a prior on *w*_*i*,*j*_6

with positive shape and rate parameters *q* and *s* [48, 49]. Note that, after taking the negative logarithm and dropping the normalizing factor 1/(*qs*^*q*^), this is equivalent to regularization using the *L*_*q*_-norm, and corresponds to a Laplacian prior if *q* = 1 or a normal prior if *q* = 2, and the parameter *s* determines the width of the distribution. The parameters *μ*_*i*,*j*_ can be used to encode additional knowledge on specific edges, by setting individual *μ*_*i*,*j*_ to nonzero values. If no prior knowledge is available, *μ*_*i*,*j*_ defaults to 0. We note that the *μ*_*i*,*j*_ can be chosen continuously depending on the expected strength of the effect and certainty in its presence. We expect signaling molecules to be "off" in the absence of any signal, and therefore use independent negative gamma priors for *p*(*w*_0,*i*_),
7

with rate and shape parameters *r*_0_ and *s*_0_, respectively. We hence assume *w*_0,*i*_ to be non-positive. This assumption can easily be replaced if signaling molecules should also be allowed to be "on" in the absence of signal, for example by using a gamma prior on the absolute value |*w*_0,*i*_|, or a normal prior on *w*_0,*i*_. Lastly, as model time is discrete and necessarily positive, we use a binomial prior *p*(*τ*_*i*_) on *τ*_*i*_,
8

with parameters *n* and *p*.

### Network inference

We could now maximize  to find the most probable network Ω underlying the data . This is reasonable if the posterior is unimodal and has a sharp, narrow optimum. However, since the optimization problem encountered for real data is typically underdetermined, several alternative models Ω often explain the data equally well, and marginal distributions for parameters show wide peaks and large confidence intervals. We therefore use Markov chain Monte Carlo (MCMC) sampling to fully characterize the posterior. The main difficulty in doing this lies in the calculation of the likelihood , which due to the required marginalization over unobserved nodes and time points quickly becomes very involved and time-consuming, compare equation (3). We therefore approximate  by simulating the dynamics of the model with parameters Ω for each knockdown , and then compare the simulation results *M*_*k*_(*t*) at each time point *t* with the experimental data *d*^*k*^(*t*). If this is repeated a large number of times, we can approximate  by the number of times we get  back in the simulation runs. By combining this with MCMC sampling, a very efficient approach for evaluation of the posterior distribution arises, that does not require an explicit computation of the likelihood [50]. In contrast to PBTN, which employed a Metropolis-Hastings based sampler, we have now implemented a far more efficient sampler in D-PBTN. We employ distributed evolutionary Monte Carlo (DEMC) [51], a sampler that combines features of a distributed genetic algorithm with MCMC sampling. In brief, DEMC starts with a population of *m* Markov chains, which are grouped into *g* subpopulations. Each individual chain describes a specific network Ω. Updates within each subpopulation are done using the genetic operators mutation and cross-over, and migration allows individual solutions to move between subpopulations. Thereafter, each chain is scored by initializing each node in the network with the values experimentally observed at time 0, and then simulating using equation (1). This is followed by a Metropolis-Hastings step to accept or reject the new state, to ensure ergodicity of the chains. A detailed description of the procedure is given in Additional file 1.

### Data simulation

**Figure 1:**
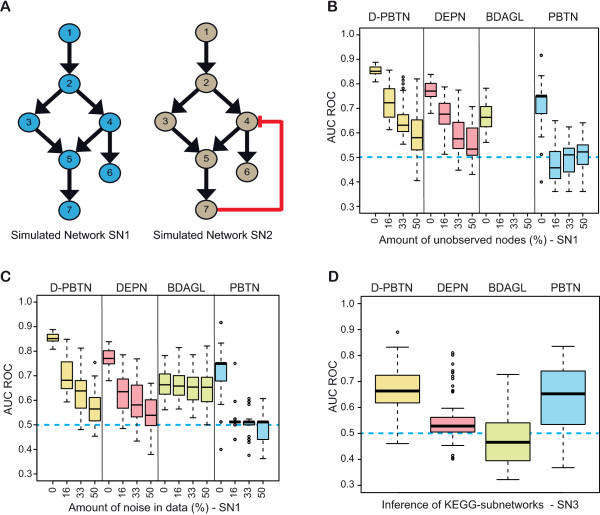
**Evaluation on simulated data. A**: The panel shows network topologies used to simulate data. *Simulated Network 1 (SN1)* is a simple feedforward network, whereas *Simulated Network 2 (SN2)* contains a negative feedback loop. **B**: Network reconstruction was performed for SN1, changing the number of unobserved proteins. Shown is the distribution of the area under the ROC curve (*AUC*_*ROC*_) of 100 replicates of simulated data sets, over the fraction of unobserved proteins. From left to right: D-PBTN, DEPN, BDAGL, PBTN. The dashed line at *AUC*_*ROC*_ = 0.5 shows expected results for random guessing. **C**: The panel shows the distribution of *AUC*_*ROC*_ values obtained for different levels of noise on SN1. Noise is introduced by switching the state of the indicated fraction of proteins in the "measured" data, thus introducing errors in the data. **D**: This panel shows performance of D-PBTN, DEPN, BDAGL AND PBTN on networks sampled from KEGG, with data simulated as described in methods. Shown are *AUC*_*ROC*_ values of100 simulated data sets, generated from ten different KEGG subnetworks.

Since for real experimental data typically no "gold standard" network is available to assess results, we used simulated data to evaluate D-PBTN. This allows it to systematically alter properties of the data, such as the inherent level of noise or amount of missing values, and to directly assess the performance impact this has. We simulated data in three different ways:

*Simulated Network 1 (SN1)* is a 7-node feedforward network, shown in Figure 1A (left). Weights *w*_*i*,*j*_ were set to 1 for edges shown in the figure, and 0 otherwise. Baseline weights were set to *w*_0,*i*_ = -0.25 to have a high probability for an unregulated protein to be in the *off* state. Data simulation for this network was performed assuming that all proteins are in the *off* state initially, except for the receptor 1. Stochastic signaling is then simulated using equation (1), updating all nodes simultaneously, and using *γ* = 10. Knockdowns were simulated by fixing targeted proteins in the *off* state. We simulated knockdowns of all individual proteins, and two combinatorial knockdowns of proteins (3,4) and (4,6). Two time points were used for network inference, the first one immediately after knockdown and activation of the receptor; the second time point after 6 simulation steps, allowing for sufficient time for the signal to propagate through the whole network.

Using the same network topology and parameters, we furthermore simulated overexpression experiments (*Simulated Network 1a, SN1a*). Overexpressed nodes were fixed to the *on* state, while we assumed all other nodes to be inactive initially. We simulated overexpression experiments of all individual nodes, as well as combinatorial overexpression of nodes (3,4) and nodes (4,6), again by running the simulation over 6 time steps as above.

*Simulated Network 2 (SN2)* extends *SN1* by a feedback loop, see Figure 1A (right). Data for *SN2* was simulated using the model proposed by Fröhlich *et al.* [34]. In contrast to *SN1*, signaling is deterministic, and noise arises only at the measurement stage. We initialized all nodes as above and assumed that a node deterministically becomes active if there are more activating than inhibiting proteins among its parents. Measurements were simulated by sampling from a normal distribution with -distributed mean for active nodes, and -distributed mean if the node was inactive. The variances of the normal distributions were drawn from an inverse scaled chi-squared distribution , as suggested by Fröhlich *et al.* [34] based on their experimental observations. Measurements were simulated immediately after the knockdown and activation, and after four and six simulation steps.

*Simulated Network 3 (SN3)* comprises a set of ten different networks randomly extracted from the KEGG database, each containing seven connected proteins, taking only protein-protein interactions into account. We simulated time-course data for 10 time points as described for *SN2* for each of the ten subnetworks. For each subnetwork, in addition to the starting point, two further randomly selected time points were included into the final data set used for network reconstruction. Reported performance results are average performances over all ten subnetworks, avoiding biasing of results towards a single topology or time interval.

### Implementation and performance evaluation

We implemented D-PBTN in *C♯*, and evaluated the approach on both simulated and real experimental data. Source code (tested on the Windows platform) and additional information is available from our website at http://www.kaderali.org/software.html.

Convergence of the Markov chains was assessed using the methods proposed by Brooks and Gelman [52], results are given in Additional file 1. Sampled networks were aggregated using halite clustering [53]. Halite is a density-based clustering method, that we use to identify clusters of similar networks from the Markov chains. Cluster representative networks were computed using the within-cluster median of *w*_*i*,*j*_.

To quantify network reconstruction performance, we performed receiver operator characteristic (ROC) and precision recall (PR) analysis. In brief, a threshold *δ* is used for discretization of edge values, where the median  of sampled values for a particular edge *w*_*i*,*j*_ is compared with *δ*. If , the edge *e*_*i*,*j*_ is assumed present in the network, , and absent otherwise, . The resulting network with edge set  is then compared against the gold standard network, and sensitivity, specificity and precision are computed for given *δ*. This is then repeated by varying *δ*, and sensitivity is plotted over specificity for different *δ* in a ROC plot, and precision over sensitivity in a PR plot. Finally, both ROC and PR curves can be summarized by computing the area under the curve (*AUC*_*ROC*_ and *AUC*_*PR*_, respectively) as single numbers summarizing performance over a wide range of threshold values *δ*. The advantage of this approach is that it is not necessary to pick a specific threshold *δ* for the discretization, which may be difficult to do as this depends on (unknown) user preferences regarding the tradeoff between false positive and false negative edges, but the analysis summarizes performance over the full range of feasible thresholds *δ*.

We first assessed performance of D-PBTN on simulated data, varying the amount of noise in the data, using different amounts of simulated data and different models for data simulation, and evaluated stability of results for changing model hyperparameters. We then assessed performance on publicly available data regarding signaling in the ERBB network. Inferred networks on real data were assessed using STRING 9.0 as reference [54], using only edges with at least 70*%* confidence in STRING. Since interactions given in STRING are undirected and unsigned, we disregarded directional information and edge sign. We compare performance of our approach with DEPN [34], the non-time-series version of our approach (PBTN) [35], the Bayesian Directed Acyclic Graph Learning tool (BDAGL) developed by Eaton and Murphy [40, 41], and with random guessing. Notably, D-DEPN could not be used on the simulated data due to the nature of the short time courses used. Results for PBTN were obtained using a modified version of the original PBTN implementation [35] that optimizes the posterior distribution instead of sampling, thus allowing for a more efficient computation on networks with a unimodal posterior. This compromise is necessary, as the original sampling-version of PBTN is too slow to allow a rigorous evaluation on networks of the size used here. Time points were used as independent measurements in PBTN. To obtain results for the DEPNs, we used greedy hill-climbing and bootstrapping with 100 bootstrap samples, as proposed by the authors of the method. The bootstrap samples were used to obtain weights on the edges, which were subjected to ROC and PR analysis. We note that DEPNs operate on equivalence classes of graphs, and can not distinguish between graphs within an equivalence class. We therefore used the transitively closed network as a representative of the full equivalence class to evaluate the DEPN results. This is in contrast to the other methods, which in principle are able to determine a unique network, provided sufficient data is given, and which we compared directly to the gold standard network. Lastly, results for BDAGL were obtained using the modified marginal likelihood method for perfect interventional data, with uniform prior.

## Results

As a first test of our method, we assessed the performance of D-PBTN on simulated data, without noise and with full observations of all nodes, using the network topology SN1. To compare performance of D-PBTN with other approaches, we used the non-time series version PBTN, DEPN and BDAGL. For D-PBTN, shape and rate parameters of the prior distribution (6) on *w* were set to *q*,*s* = 1, respectively, corresponding to a standard L1 regularization of the edge weights as the simplest conceivable setting. Rate and shape parameters of the negative gamma prior on *w*_0,*i*_ were set to *r*_0_ = 8, *s*_0_ = 16, resulting in a moderate deactivation of unregulated proteins, and a binomial prior with *n* = 20,*p* = 0.3 was used for *τ*, giving an expected value of *τ* of 6 time steps. The stochasticity parameter *γ* was set to 1.8, corresponding to an average level of noise in the data. Three sub-populations with four Markov chains were used with 10^8^ steps each and a burn-in of 5,000 steps. Parameters for PBTN were set accordingly, using the two time points as independent replicate measurements. Results for all four approaches are shown in Table 1, values shown are median values of the *AUC*_*ROC*_ and *AUC*_*PR*_ measures over 100 replicate simulations. With no noise and no missing values, D-PBTN shows superior performance over the other three approaches on the simulated data. Notably, the comparison between D-PBTN and PBTN shows the added value of the temporal information, indicating that while a significant part of the information in the data comes from the knockdowns, even short time courses with just 2 time points are of added value. BDAGL shows weaker results than the other three approaches, both in terms of the *AUC*_*ROC*_ and *AUC*_*PR*_, possibly due to a significantly different underlying mathematical model.

**Table 1:** **Performance comparison of D-PBTN, PBTN, DEPN and BDAGL on simulated data**

	***AUC***_***ROC***_								
Network	SN1				SN2				SN3
Noise	0%	16 %	33%	50%	0%	0%	0%	0%	0%
Missing	0%	0%	0%	0 %	16 %	33%	50%	0%	0%
D-PBTN	0.85	0.68	0.64	0.56	0.72	0.63	0.58	0.78	0.66
PBTN	0.75	0.51	0.51	0.51	0.46	0.51	0.52	0.75	0.65
DEPN	0.77	0.63	0.58	0.54	0.68	0.58	0.53	0.60	0.53
BDAGL	0.66	0.66	0.65	0.65	-	-	-	0.75	0.47
	***AUC***_***PR***_								
**Network**	**SN1**				**SN2**				**SN3**
Noise	0%	16 %	33%	50%	0%	0%	0%	0%	0%
Missing	0%	0%	0%	0 %	16 %	33%	50%	0%	0%
D-PBTN	0.80	0.48	0.43	0.33	0.52	0.41	0.36	0.58	0.45
PBTN	0.79	0.58	0.58	0.58	0.18	0.20	0.23	0.75	0.58
DEPN	0.69	0.41	0.33	0.29	0.39	0.35	0.31	0.41	0.18
BDAGL	0.19	0.19	0.19	0.20	-	-	-	0.53	0.28

Shown are achieved values of the area under the ROC curve (*AUC*_*ROC*_) and the area under the PR curve (*AUC*_*PR*_). Values shown are median values over 100 iterations. Inference was performed on the SN1 data, with data with different levels of noise and missing values, on the SN2 data including a negative feedback loop, and on the SN3 (KEGG) data set. Note that BDAGL cannot handle missing values. The upper part of the table shows the area under the ROC curve, while the lower part of the table shows the area under the PR curve.

### Effect of missing data

We next assessed the effect of missing observations on network inference, using the SN1 data. Results are shown in Figure 1B for *AUC*_*ROC*_ and Additional file 2: Figure S1 for *AUC*_*PR*_, and are summarized in Table 1. Note that BDAGL could not be used in this comparison, as the method cannot handle missing values. We randomly selected 16%, 33% and 50% of the nodes, and completely removed the corresponding simulated observations for these nodes before carrying out the network inference. Model parameters were set as described above. Computation took approximately 7.4 hours for D-PBTN on a 3 GHz Intel 64-bit processor (single-threaded). For comparison, runtime for DEPN was only around 30 minutes. Regarding quality of the inferred networks, D-PBTN shows superior performance over DEPN, both with respect to *AUC*_*ROC*_ and *AUC*_*PR*_. PBTN did not perform better than random guessing on all runs with missing values (*AUC*_*ROC*_ ≈ 0.5). Both D-PBTN and DEPN show a decrease in the quality of the inferred network with increasing amounts of missing observations. In spite of this, even with 50% of missing data, both approaches still perform significantly better than guessing (both *p* < 0.0001, Welch’s t-test on *AUC*_*ROC*_ values). Median *AUC*_*ROC*_ decreased from 0.85 to 0.58 (D-PBTN), 0.77 to 0.53 (DEPN) and 0.75 to 0.52 (PBTN) when comparing the 0% to the 50% missing value performance. For each considered amount of missing data, D-PBTN outperformed DEPN (0% missing: *AUC*_*ROC*_ 0.85 vs. 0.77, *p* < 0.0001; 16% missing: *AUC*_*ROC*_ 0.72 vs. 0.68, *p* < 0.0001; 33% missing: *AUC*_*ROC*_ 0.63 vs. 0.58, *p* < 0.0001; 50% missing: *AUC*_*ROC*_ 0.58 vs. 0.53, *p* = 0.0029); results are comparable for *AUC*_*PR*_, see Table 1.

### Effect of noise

We next tested the effect of noise on inference performance, see Figure 1C for *AUC*_*ROC*_, Additional file 3: Figure S2 for *AUC*_*PR*_, and the summary in Table 1. To simulate noise, we randomly changed individual measurements in SN1 from 0 to 1 and vice versa. Network inference was performed on the resulting data (with no missing values), with parameters as described above, and resulting in comparable running times. All four approaches showed decreasing performance for increasing levels of noise, with median *AUC*_*ROC*_ decreasing from 0.85 (0% noise) to 0.56 (50% noise) for D-PBTN, from 0.77 to 0.54 for DEPN, from 0.75 to 0.51 for PBTN, and from 0.66 to 0.65 for BDAGL. D-PBTN was the best performing method using *AUC*_*ROC*_ as performance measure, whereas PBTN was not better than guessing, with median *AUC*_*ROC*_ = 0.51 already at 16% noise. In contrast to this, we observed good *AUC*_*PR*_ performance of PBTN, with *AUC*_*PR*_ = 0.58 at all noise levels. Directly comparing D-PBTN with the non-time-series version PBTN, we observed that *AUC*_*ROC*_ results for PBTN were inferior to D-PBTN at all noise levels, whereas PBTN showed better *AUC*_*PR*_ on data with higher levels of noise. This is a very intersting observation, which we discuss further in the Discussion section below. BDAGL showed very robust performance for increasing noise levels, consistently yielding low, but stable *AUC*_*ROC*_ ≈ 0.65, but with very low *AUC*_*PR*_ ≈ 0.19. We observed a further performance breakdown for BDAGL when noise levels increased beyond 50% (data not shown). Finally, comparing D-PBTN with DEPN, even with 50% of the data wrong, both approaches are significantly better than random guessing (both *p* < 0.0001), and D-PBTN outperforms DEPN for all noise levels (0% noise: *AUC*_*ROC*_ 0.85 vs. 0.77, *p* < 0.0001; 16% noise: *AUC*_*ROC*_ 0.68 vs. 0.63, *p* < 0.0001; 33% noise: *AUC*_*ROC*_ 0.64 vs. 0.58, *p* = 0.0014, 50% noise: *AUC*_*ROC*_ 0.56 vs. 0.54, *p* = 0.0052); results for *AUC*_*PR*_ were qualitatively equivalent, see Additional file 3: Figure S2 and Table 1.

### Overexpression experiments

As a further test of the method, we assessed the use of overexpression data with D-PBTN, using the SN1a data set. Model parameters were chosen as for the knockdown experiments above.

Results were comparable to results obtained using the knockdown data, with *AUC*_*ROC*_ = 0.88 (Knockdown: 0.85) and a *AUC*_*PR*_ = 0.79 (Knockdown: 0.80) for D-PBTN. For comparison, we also used DEPN with the overexpression data. This resulted in an *AUC*_*ROC*_ = 0.63 and *AUC*_*PR*_ = 0.62, both inferior to the D-PBTN results. We note that neither BDAGL nor PBTN in its original implementation are able to handle overexpression data, and could therefore not be included in the comparison.

### Effect of network topology and model assumptions

Data SN1 were simulated using the model underlying our approach. Results may thus favor D-PBTN and PBTN. Furthermore, artificial topologies may not be representative of real biological networks. We therefore sampled "real" subgraphs from the KEGG database, and simulated data using a different model (section "Data simulation"). The obtained data set SN3 was used to assess performance of D-PBTN, PBTN, BDAGL and DEPN. D-PBTN prior and MCMC and PBTN parameters were chosen as above, except for the parameter *p* of the binomial prior on *τ*, which was set to *p* = 0.2, resulting in a slightly smaller expected value for *τ*. Results for BDAGL and DEPN were obtained as described above.

Figure 1D and Additional file 4: Figure S3 show *AUC*_*ROC*_ and *AUC*_*PR*_ results obtained, respectively, and all results are summarized in Table 1. Median *AUC*_*ROC*_ values achieved were 0.66 (D-PBTN), 0.65 (PBTN), 0.53 (DEPN) and 0.47 (BDAGL); Median *AUC*_*PR*_ values were 0.45 (D-PBTN), 0.58 (PBTN), 0.18 (DEPN) and 0.28 (BDAGL). *AUC*_*ROC*_ results for D-PBTN, PBTN and DEPN are statistically significantly better than guessing (*p* < 0.0001). D-PBTN outperformed DEPN both with respect to *AUC*_*ROC*_ and *AUC*_*PR*_ (Welch two sample t-test, both *p* < 0.0001). We observed that DEPN had particular difficulties when measured time points were far apart in time, with several unobserved intermediate steps between them. D-PBTN, which in contrast to DEPN were specifically designed for time course data, did not exhibit this problem. Notably, the non time-series method PBTN showed only marginally inferior *AUC*_*ROC*_ than D-PBTN (median *AUC*_*ROC*_ = 0.66 D-PBTN vs. 0.65 PBTN, *p* = 0.051), but superior performance in terms of median *AUC*_*PR*_ (0.45 D-PBTN vs. 0.58 PBTN). However, in contrast to D-PBTN, PBTN results are characterized by high variability (see Additional file 4: Figure S3, inter quartile range (IQR) , IQR ), and mean *AUC*_*PR*_ values are very similar between the two approaches (0.457 D-PBTN vs. 0.46 PBTN). A statistical test comparing the *AUC*_*PR*_ values from the 100 replicate runs of the two approaches shows no significant difference in performance measured by *AUC*_*PR*_ (*p* = 0.9156, Welch two sample t-test).

We next evaluated the effect of negative feedback loops on network inference, using data set SN2. This is a difficult problem, as the feedback can only be inferred from the temporal evolution of the network. Parameters for Markov chains and prior were set as above, using 5 subpopulations with 3 chains each in sampling. This resulted in a running time of 16 hours (D-PBTN). Using the full data set, D-PBTN achieved a median *AUC*_*ROC*_ of 0.78 and median *AUC*_*PR*_ of 0.58; DEPNs achieved a median *AUC*_*ROC*_ of 0.60 and median *AUC*_*PR*_ of 0.41. Median *AUC*_*ROC*_ performance values of PBTN and BDAGL were both 0.75, and were superior to DEPN, but inferior to D-PBTN. In line with results on data set SN3, we observed good *AUC*_*PR*_ performance of the non time-series method PBTN (median *AUC*_*PR*_ 0.75 PBTN vs. 0.58 D-PBTN), however, in contrast to D-PBTN, PBTN could not infer the feedback loop between nodes 7 and 4.

**Figure 2:**
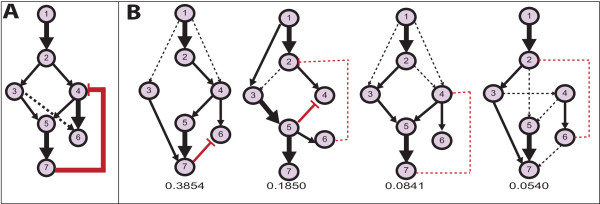
**Inference of negative feedback cycles.** The Figure shows reconstructed network topologies on simulated data SN2, using the full data set (panel **A**) and partial data with observations only for proteins 6 and 7 (panel **B**). In the latter case, no unique network can be identified anymore. Clustering of sampled *w* groups topologies according to structural similarity. Only one cluster arises for the complete data (panel **A**), whereas four major different clusters arise on partial data (panel **B**). Shown are median networks from the four clusters, together with cluster probabilities computed as the fraction of networks in the cluster to the total number of sampled networks. Edge thickness indicates strength of support for the edge within the cluster (thick solid: >80*%* of samples within cluster, thin solid: >60*%*, dashed: >40*%* support), black lines indicate activations, red lines inhibitions.

Additional file 5: Figure S4A shows the distribution of edge weights obtained with D-PBTN, indicating that the posterior distribution (4) is unimodal. Using only observations of proteins 6 and 7, unique networks can no longer be recovered (Additional file 5: Figure S4B). Probabilities for individual network topologies or even edges can be computed based on the sampled points, and can then be used e.g. to design experiments to resolve the true underlying network (Figure 2).

### Robustness with respect to model hyperparameters

Since our model contains parameters to be set by the user, we next analyzed how robustly networks were inferred for varying model hyperparameters, using data set SN1. We varied *γ* in equation (1) by up to ±50*%*. For changes of *γ* by the maximum change of ±50*%* tested, the change observed in inference performance was a decrease of 23.8% for *AUC*_*ROC*_, and 26.5% for *AUC*_*PR*_ (Additional file 6: Figure S5). Of note, even for a change by 50%, D-PBTN still performed significantly better than random guessing (*AUC*_*ROC*_ 0.643, *AUC*_*PR*_ 0.593). We furthermore studied the influence of changing the prior hyperparameters *q* and *s* of the prior *p*(*w*_*i*,*j*_) (equation (6)), changing them simultaneously by up to ±20*%* (Additional file 7: Figure S6, panel A and B), or individually by up to 50% (supplementary Figure S6, panel C and D). This resulted in a maximum change of 24.7% in *AUC*_*ROC*_ and 22.3% in *AUC*_*PR*_ for individual changes of *q* by 50%, decreasing *AUC*_*ROC*_ to 0.64 and *AUC*_*PR*_ to 0.59, and changes by 11.7% and 12.6% for *AUC*_*ROC*_ and *AUC*_*PR*_, respectively, for changes of *s* by 50%. We then tested the influence of changing the parameters *n* and *p* of the binomial prior on *τ* by 50%, observing only a relatively minor impact of these parameters on performance (changes in *AUC*_*ROC*_ between 0.797 and 0.856, changes in *AUC*_*PR*_ between 0.719 and 0.824). A more significant influence was observed for the parameters *r*_0_ and *s*_0_ of the negative gamma prior on *w*_0_, which influences the baseline activity level of unregulated nodes, see Additional file 8: Figure S7. Overall, robustness analysis indicates that inference is reasonably stable for varying hyperparameters; still, some care should be exercised in choosing values.

### Inferring ERBB-mediated signal transduction

**Figure 3:**
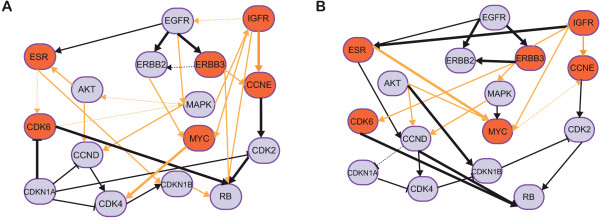
**Reconstructed ERBB-mediated signaling network.** The plots show inferred network topologies for reverse phase protein array data acquired after stimulation of ERBB signaling and knockdowns of proteins involved in ERBB signaling. True positive edges, using high-confidence interactions in the STRING database as reference, are shown as solid black lines, newly predicted edges are shown as orange lines. True and false negatives are not depicted for the sake of readability of the plot. Edge thickness indicates strength of support for the edge (thick solid: >80*%* of samples, thin solid: >60*%*, dashed: >40*%* support). **A**: Network inferred without using any prior knowledge, **B**: Network inferred using the literature interactions reported by [34] as prior knowledge for network inference.

To test D-PBTN on real data, we used publicly available data from Fröhlich *et al.* [34], regarding 16 proteins involved in ERBB signaling. The data comprises 3 replicate measurements for 10 of the 16 proteins, measured after 16 different knock-downs using RNA interference, including 3 combinatorial knockdowns. Measurements were taken at two different time points, directly before stimulation with EGF, and after 12 hours of stimulation, using reverse phase protein arrays. We discretized the normalized data using the midpoint of the negative and positive control medians as discretization threshold. Network inference was performed in 10 subpopulations with 5 chains each, sampling over 10^6^ steps with a burn-in of 8,500 steps. Parameters *q* and *s* were set to 0.35 and 14, respectively, corresponding to a fairly strict regularization. We set the stochasticity parameter *γ* to *γ* = 8, to obtain a slightly more deterministic behavior of the model than in case of the simulation study. Rate and shape parameters of the gamma prior *p*(*w*_0,*i*_) were set to *r*_0_ = 10,*s*_0_ = 17, keeping unregulated nodes in the "off" state, and parameters of the binomial prior *p*(*τ*) were set to *n* = 20 and *p* = 0.4, allowing for a relatively large number of model steps between the experimental measurements. Computation time took approximately 1590 minutes, or roughly 26.5 hours. Clustering of the 10^6^ sampled weight vectors *w* shows only a single cluster with high probability, indicating a unimodal posterior. To obtain a single network for visualization purposes and further biological discussion of inferred edges, we used a threshold *δ* = 0.65× max|*w*_*i*,*j*_| for network discretization, including only edges with sample median  in the further evaluation - in words, edges were only included if the median of all points sampled for the corresponding edge was at least 65% of the maximum edge value sampled. This results in a network with comparable number of edges as the gold standard network shown in Additional file 9: Figure S8, constituting a tradeoff between false positive and false negative edges. We compared results obtained using D-PBTN with the non-time series version PBTN [35], as well as DEPN. Results for DEPN were taken from the original publication [34], the authors do not report AUC ROC and PR values for their method. Figure 3A shows the resulting network using D-PBTN, and Table 2 summarizes the results obtained using D-PBTN, PBTN and DEPN. Notably, without inclusion of any additional prior knowledge, both D-PBTN and DEPN show similar specificity and accuracy, but D-PBTN is both more sensitive and has higher precision than DEPN. The dynamic version D-PBTN outperform PBTN in all performance measures used, showing the additional value of the temporal information in the data when using this method.

**Table 2:** **Performance comparison ERBB signaling network**

	Sensitivity	Specificity	Accuracy	Precision	***AUC***_***ROC***_	***AUC***_***PR***_
D-PBTN	0.44	0.83	0.75	0.41	0.72	0.64
PBTN	0.20	0.77	0.66	0.20	0.52	0.31
DEPN	0.26	0.86	0.73	0.33	not avl.	not avl.
D-PBTN (+Prior)	0.59	0.90	0.84	0.62	0.78	0.70
DEPN (+Prior)	0.59	0.87	0.81	0.55	not avl.	not avl.
Prior network	0.48	0.87	0.79	0.5	-	-

Achieved sensitivity, specificity, accuracy and precision for the inference of ERBB signaling [34]. Shown are results of D-PBTN, PBTN and DEPN, with and without prior information (+Prior). The last row shows results of the prior network alone. Network inference was assessed using STRING as gold standard. Results for DEPN were taken from Froehlich et al. [34], authors do not report *AUC*_*ROC*_ or *AUC*_*PR*_ values.

The inferred network (without using a network prior) is shown in Figure 3A, and displays significant cross-talk in ERBB-signaling. At the surface receptor level, D-PBTN predicts cross-talk between EGFR, ESR and IGFR, as recently confirmed experimentally [55]. We furthermore predict a direct interaction between AKT and MAPK, linking the corresponding pathways. In fact, involvement of MAPK signaling in AKT activation was recently reported [56]. At the transcription factor level, D-PBTN predicts an interaction between CDK4 and MYC, in line with observed expression changes of CDK4 and CDK6 following MYC degradation [57].

**Figure 4:**
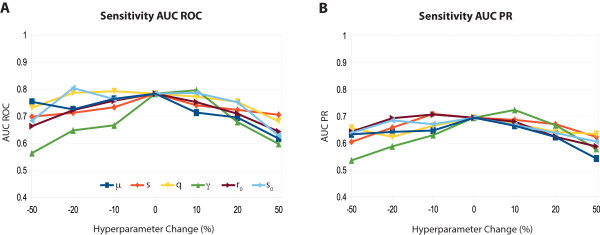
**Hyperparameter sensitivity analysis ERBB network.** The Figure shows sensitivity of the *AUC*_*ROC*_ (panel **A**) and *AUC*_*PR*_ (panel **B**) of the ERBB network (with literature prior) with respect to changes in model hyperparameters. Model parameters *μ*, *s*, *q*, *γ*, *r*_0_ and *s*_0_ where changed by up to ±50*%*, and inference was repeated for each parameter choice. Shown are resulting values for the area under the receiver operator characteristic (*AUC*_*ROC*_) and the area under the precision-recall (*AUC*_*PR*_) curves. The analysis indicates that inference results are reasonably robust to changes in model hyperparameters, with the stochasticity parameter *γ* being the most critical parameter for inference performance.

Inference quality improves dramatically when prior knowledge is provided. We used the literature network presented in Fröhlich *et al.* [34] as prior knowledge, with *q* = 2 and *s* = 1, and *μ*_*i*,*j*_ = +3 in case of an activation, -3 in case of an inhibition, or 0 in case of no regulation in the prior network. We note that this prior network alone yields superior results than D-PBTN without any prior knowledge (compare Table 2), emphasising the importance of using a network prior on this data set. Network inference was performed as above, resulting in the network depicted in Figure 3B. Obtained results are superior to both, using prior knowledge alone, as well as using only the experimental data (Table 2). To assess robustnes of these inference results with respect to model hyperparameters, we furthermore performed a sensitivity analysis by modifying model hyperparameters individually by up to ±50*%*, and evaluated changes in resulting AUC ROC and AUC PR, compare Figure 4A and B. This analysis indicated that results are relatively stable, with the most influential parameter being the stochasticity parameter *γ*.

We evaluated predictions using IPA (Ingenuity), and could find additional evidence for most of the predicted edges: D-PBTN predicts regulations of IGFR, MAPK and CDK6 by ERBB3. Indeed, binding of ERBB3 and IGFR has been shown in lysate from SkBr3 cells resistant to trastuzumab by immunoprecipitation [58]. Similarly, there is experimental evidence for an activation of MAPK by ERBB3, as we predicted [59, 60]. The inferred activation of CDK6 by ERBB3 to our knowledge has not been reported before, and may warrant further experiments to confirm or falsify this prediction. Some evidence also exists for the predicted strong activation of MYC by ESR. Both proteins have been shown by affinity chromatography to bind to one another [61], and blocking of ESR1 was recently shown to decrease MYC expression [62]. Similarly, activation of AKT increases CCND and MYC expression [63, 64], and a regulation of CCNE by MYC [65, 66] and the activation of CCND by MAPK [67] have been demonstrated experimentally. Interestingly, D-PBTN predicts an activation of MYC and CCNE by IGFR. Both interactions to our knowledge have not been previously reported, and may be interesting candidates for experimental validation.

## Discussion

In this manuscript, we present D-PBTN, a novel method to infer signal-transduction networks from time course perturbation data. We evaluate D-PBTN in an extensive simulation study, and demonstrate its application on experimental data regarding ERBB signaling. The simulation study shows stable inference results in light of missing data and noise, and reasonable robustness with respect to model hyperparameters. On the ERBB application, we have run the inference with and without a network prior, and provide both results for comparison in Figure 3. While the overall topology is similar whether or not a prior network is used, there are significant differences in individual edges. The quality of network inference with a network prior clearly depends on the quality of the prior used as well as on the amount and quality of experimental data available, we therefore recommend to carefully evaluate the effect a given network prior has. In our example, there is experimental evidence for many of the predicted interactions, both from the run with and without network prior, directly confirming the quality of the inference. Furthermore, in addition to confirming known interactions, D-PBTN predicts several novel edges, which may be interesting candidates for experimental validation.

Running time remains a major concern with D-PBTN, mainly due to the time required for sampling from the posterior distribution. Each single sampling step requires running time *O*(*KTN*^2^), where *K* is the number of perturbations, *T* is the number of (equidistant) time steps, and *N* is the number of nodes in the network. This can be easily seen from the likelihood function (2), which essentially iterates over all time points, perturbations and nodes in the network, and computes equation (1) in each step, which again iterates over all nodes. Running time for the likelihood dominates the time required for the prior distribution, hence the total running time for the posterior is *O*(*KTN*^2^). Unfortunately, the shape and complexity of the likelihood function, and thus the number of sampling steps required to sample from the posterior *p*(*D*|Ω) (both to reach the stationary distribution of the chain, and number of steps required to sufficiently traverse the support of the posterior to get a good representation of the distribution in the sample), also depends on *K*, *T* and *N* in a nontrivial way, and the choice of required sampling steps (with associated impact on total running time of the algorithm) is a nontrivial problem. Clearly running time increases linearly with the number of sampling steps, but how exactly the minimum number of sampling steps required depends on *K*, *T* and *N* is unclear.

While the present implementation of D-PBTN is a non-parallel implementation, evolutionary MCMC is straightforward to parallelize, allowing for a very efficient parallel implementation of the sampler. In practical terms, running time on the SN1 example (7 nodes) was approximately 7.4 hours with the present, non-parallelized version of the algorithm, and computations much beyond the size of the ERBB network with its 16 nodes appear computationally prohibitive without further parallelization. An efficient parallel implementation on a larger compute cluster in principle should make D-PBTN suitable also for "larger" network inference problems with several dozen, possibly up to a few hundred nodes, but this is clearly not an approach that is suitable for networks with thousands of proteins, let alone proteome-wide models.

As with all Bayesian approaches, prior distributions and prior hyperparameters impact results, and need to be carefully chosen. Reasonable values for some of the parameters can be estimated from biological expectations, for example the expected level of "connectedness" in a network can be used to estimate parameters *q* and *s* of the prior on *w*, or information about the speed of signal transduction in a network could be used to estimate hyperparameters for the prior on *τ*. However, still a significant amount of "gut feeling" and experience are required to get good estimates. While our simulation study indicates reasonable stability with respect to parameter changes, a possible extension of our work is to employ empirical Bayes’ approaches to set model hyperparameters [17, 18], or, granted enough experimental data and compute time are available, crossvalidation-schemes could be used for hyperparameter estimation.

The comparison between D-PBTN and the non time-series version PBTN on the different simulated data sets gave some very interesting results. While D-PBTN outperformed PBTN on all data sets when *AUC*_*ROC*_ was used as performance measure, PBTN yielded higher median *AUC*_*PR*_ than D-PBTN on datasets with high levels of noise, or data simulated using a different underlying mathematical model. *AUC*_*PR*_ in contrast to *AUC*_*ROC*_ is less influenced by an unequal distribution of class labels (as occurs in sparse networks), the superior results of PBTN over D-PBTN under certain conditions are therefore of relevance for the choice of inference method. It can be shown that if a ROC curve for method A dominates a ROC curve for method B, then also the corresponding PR curve for method A dominates the PR curve for method B, and vice versa [68]. The inverted ranking of D-PBTN and PBTN in *AUC*_*ROC*_ and *AUC*_*PR*_ implies that the ROC curves (and by the same argument the PR curves) of D-PBTN and PBTN do not dominate one another. Rather, there are certain domains in the ROC and PR plots where one or the other method becomes better, and correspondingly the curves for D-PBTN and PBTN intersect. This in particular occured in our simulation study for the data with high levels of noise. It is tempting to speculate that under conditions with many "wrong" data points, focusing on a stable steady state (as done with PBTN) may have advantages over trying to capture the full dynamics of the system, as D-PBTN does. This point will require further research in the future, to determine what exactly determines which of the two approaches is superior under given conditions, and to provide guidelines for method selection. On the other hand, if the interest is in feedback loops and cycles in the networks, PBTN should not be used, as this approach cannot infer cyclic networks. Interestingly, on the ERBB application example, D-PBTN clearly outperformed PBTN both with respect to *AUC*_*ROC*_ and *AUC*_*PR*_.

## Conclusion

In this manuscript, we have developed a new method to infer signal transduction networks from time course perturbation data. Based on relatively few time points after a large number of different perturbations, our method is able to reconstruct the underlying signaling network with high accuracy. The mathematical approach we employ is based on dynamical Bayesian networks, and assumes discrete states and time steps, at which signaling molecules are either "on" or "off". The approach is therefore suitable to experiments where protein activation or protein expression is completely or almost completely switched off, such as with RNAi knockdowns.

Several related approaches exist. Our work extends PBTN by explicitly taking time into account. In contrast to PBTN, the method can thereby infer feedback and feedforward loops. Provided sufficient time-resolved measurements after (possibly combinatorial) knockdowns are available, a unique network can be inferred using D-PBTN. This is in contrast to methods such as PBTN, DEPN and Nested Effects Models (NEMs), which only return equivalence classes or single networks out of an equivalence class. In this regard, our method is comparable to dynamic DEPNs and dynamic NEMs, which also explicitly take time course data into account. However, D-NEMs have been developed for "effect" observations after knockdowns, which only provide very indirect information about the underlying network. D-DEPNs, on the other hand, require long time series and work most efficiently if a relatively small number of perturbations are carried out. The typical experimental scenario encountered in practice is different, comprising short time courses after a large number of perturbations. D-PBTN have explicitly been developed for this situation. Due to the Bayesian approach pursued, prior biological knowledge can easily be integrated into D-PBTN inference, and marginalization over unobserved proteins or time points is straightforward and very efficient due to the likelihood simulation.

We employ an MCMC approach to sample from the posterior distribution of models given the experimental data, which allows it to compute probability distributions over alternative network topologies. This is particularly useful if multiple models explain the data equally well, and can be used to design additional experiments to then distinguish further between high-scoring topologies. Using halite clustering, similar models can be grouped together and probabilities estimated from the MCMC samples.

Overall, D-PBTN is a powerful approach for network inference when time-resolved measurements of the response of a dynamical system after perturbation are available. Under these conditions, the approach outperforms other state-of-the-art methods. Most approaches available in the literature consider either changes in steady state levels after perturbation, or the temporal dynamics of an unperturbed system for network inference. D-PBTN makes use of both the dynamical aspects and the perturbation effects in network reconstruction. We present an extensive simulation study to evaluate the approach, showing stable performance under varying conditions of noise and missing values in the data, and we demonstrate the application of D-PBTN to infer signaling networks in the ERBB system. Here, our approach both reconfirms known interactions, as well as suggesting some novel edges as candidates for further experimental study.

## Availability and requirements

**Project name:** Dynamic Probabilistic Threshold Networks

**Project home page:**http://www.kaderali.org/software/dpbtn.html.

**Operating system(s):** Tested on Microsoft Windows

**Programming language:** C#

**Other requirements:** None

**License:** GNU GPL

**Any restrictions to use by non-academics:** None

## Electronic supplementary material

Additional file 1: **Supplementary material.** Additional file one contains supplementary text with additional information. (PDF 233 KB)

Additional file 2: **Supplementary Figure S1.** Effect of the amount of unobserved proteins on inference performance (SN1 reconstruction). Shown is the distribution of the area under the precision recall curve (*AUC*_*PR*_) for 100 replicates of simulated data sets, over the fraction of unobserved proteins, for D-PBTN, DEPN, BDAGL and PBTN on dataset SN1. The dashed line shows expected *AUC*_*PR*_ results for random guessing. (EPS 672 KB)

Additional file 3: **Supplementary Figure S2.** Effect of noise on inference performance (SN1 reconstruction). The Figure shows the distribution of *AUC*_*PR*_ values obtained for different levels of noise on SN1, for D-PBTN, DEPN, BDAGL and PBTN. The dashed line shows expected *AUC*_*PR*_ results for random guessing. (EPS 681 KB)

Additional file 4: **Supplementary Figure S3.** Inference performance on the reconstruction the KEGG networks. This Figure shows performance of the D-PBTN, DEPN BDAGL and PBTNapproaches on subnetworks taken from the KEGG database, with data simulated as described in methods. Shown are *AUC*_*PR*_ values of 100 simulated data sets, generated from ten different KEGG subnetworks. The dashed line shows expected *AUC*_*PR*_ results for random guessing. (EPS 606 KB)

Additional file 5: **Supplementary Figure S4.** Inference of negative feedback cycles: Bean-plot showing the distributions of inferred edge weights. A unimodal distribution is observed with the full data (panel **A**), whereas no unique network can be identified when only observations of two downstream proteins are given. (EPS 5 MB)

Additional file 6: **Supplementary Figure S5.** Effect of model parameters on performance: The Figure shows the effect of varying the stochasticity parameter on the AUC values (*AUC*_*ROC*_ and *AUC*_*PR*_). Network inference has been done using data simulated from *Simulated Network 1*. *AUC*_*ROC*_ values are shown as red squares, *AUC*_*PR*_ values are shown as blue circles. (EPS 1 MB)

Additional file 7: **Supplementary Figure S6.** Effect of model parameters on performance: The Figure shows the effect of varying the rate and shape parameter *q* and *s* of the prior *p*(*w*) on the AUC values. Network inference has been done using data simulated from *Simulated Network 1*. Panels **A** and **B**: Effect of simultaneous changes of *q* and *s* by up to 20% on *AUC*_*ROC*_ and *AUC*_*PR*_. Panels **B** and **C**: Effect of changing parameters *q* and *s* individually by up to 50%. (EPS 2 MB)

Additional file 8: **Supplementary Figure S7.** Effect of model parameters on performance: The Figure shows the effect of varying the rate and shape parameters *r*_0_ (Panel **A**) and *s*_0_ (Panel **B**) of the prior on the bias weight *w*_0_ by up to 50*%*. Network inference has been done using data simulated from *Simulated Network 1*. *AUC*_*ROC*_ values are shown as red squares, *AUC*_*PR*_ values are shown as blue circles. (EPS 485 KB)

Additional file 9: **Supplementary Figure S8.** Experimental reference network extracted from STRING for ERBB network inference. (PNG 1 MB)
