# Supplementary material for: Dynamic probabilistic threshold networks to infer signaling pathways from time-course perturbation data
Source: BMC Bioinformatics. 2014 Jul 22;15(1):250. doi: 10.1186/1471-2105-15-250 (PMC4133630; doi:10.1186/1471-2105-15-250)
Supplement: Supplementary file 1 — Additional file 1: Supplementary material. Additional file one contains supplementary text with additional information. (PDF 233 KB) [file 12859_2013_6530_MOESM1_ESM.pdf]

Dynamic Probabilistic Threshold Networks to Infer Signaling Pathways from Time-Course Perturbation Data
Supplementary Material

Narsis A. Kiani and Lars Kaderali
Institute for Medical Informatics and Biometry, Medical Faculty Carl Gustav Carus, Technische Universität Dresden, Fetscherstr. 74, 01307 Dresden, Germany

May 16, 2014

1 Implementation details

The following pseudecode describes the sampling algorithm used to sample from the posterior distribution:

Algorithm 1 Network Inference by sampling from $p(\Omega|\mathcal{D})$

Initialize population of m Markov chains with random parameters $\Omega^c(0) = (\mathcal{W}^c, w_0^c, \tau^c)$ for $c = 1, \ldots, m$
$z \leftarrow 1$
repeat
  for each chain c **do**
    $\bar{\Omega}^c(z) \leftarrow$EVOLVE$(\Omega^c(z-1))$
    for each knockdown experiment $k = 1 \rightarrow K$ **do**
      Initialize nodes $\mathcal{V}^c(0) = \{v_1^c(0), \ldots, v_N^c(0)\}$
      for each measured time point $t = 1 \rightarrow T$ **do**
        $\mathcal{V}^c(t) \leftarrow$ SIMULATE$(\mathcal{V}^c(t-1), \bar{\Omega}^c(z), \mathcal{K}_k)$
      end for
      if $\forall i, t : v_i^c(t) = d_i^c(t)$ **then**
        $\zeta \leftarrow \min\{1, \mathrm{p}(\bar{\Omega}^c(m))/\mathrm{p}(\Omega^c(m-1))\}$
        Accept $\Omega^c(m) \leftarrow \bar{\Omega}^c(m)$ with probability ζ
        otherwise stay at $\Omega^c(m) \leftarrow \Omega^c(m-1)$
      else
        $\Omega^c(m) \leftarrow \Omega^c(m-1)$
      end if
    end for
  end for
  $z \leftarrow z + 1$
until enough points sampled

1.1 Evolutionary algorithm

We have used an evolutionary algorithm, DEMC, to speed up the convergence and efficiency of sampling from the posterior distribution. DEMC starts with a population of m Markov chains, which are grouped into g subpopulations. Updates of all chains in each subpopulation are done using genetic operators. Genetic operations allowed on the chains are mutation, crossover and migration. We proposed new functions for mutation and cross over which are suitable for network reconstruction. We used the migration operator as it was suggested in (Hua and Tsui, 2010).

1. Mutation: Add random mean-zero normal noise with standard deviation σ_s to one randomly chosen edge. Furthermore, add mean-zero normal noise with standard deviation σ_w to all other edges with probability $1/m^2$.

2. Crossover: Replace edges between two solutions. This operation randomly picks M edges from two networks in the same subpopulation and exchanges them, where M is randomly chosen between 0 and m^2.

3. Migration: Send randomly selected individuals as migrant in each subpopulation to the next subpopulation (Hua and Tsui, 2010).

To determine the order of subpopulations while applying the migration operator, we make a migration cycle. We choose uniformly u subpopulations, the first chosen subpopulation is the first in the migration cycle and the next one in the cycle is the next chosen subpopulation. Finally to complete the circle, the selected individual in the last subpopulation migrates to the first subpopulation. The current population, $X(t) = \{x^1(t), \cdots, x^m(t)\}$ is updated by the following iterative procedures as suggested by Hua and Tsui (2010):

1. Generate $X(t+1)$ with probability of p_m by applying migration operator, otherwise go to step 2

2. Generate $x^i(t+1)$ with probability of q_m by applying mutation operator, otherwise produce $x^i(t+1)$ by applying the crossover operator.

Each chain within each subpopulation may be mutated independently. The crossover operation is repeated iteratively until a proportion of q_c chains are updated. A discrete half normal distribution with parameter $\theta = 1$ was used for mutations on time (τ). We set $p_m = 0.1$, $q_m = 0.4$, $q_c = 0.8$ and the length of the migration cycle u was set to the number of subpopulations, $u = g$.

1.2 KEGG subnetworks

To evaluate the performance of the methods without biasing the assessment in favor of a single topology or time course interval and on networks with realistic characteristics, we extracted ten different networks from the following KEGG pathways:
KEGG IDs:(4010,4012,4020,5203,4110,4210,4062,4060,4630,4310)
We then randomly selected 7 genes from each network using the package KEGGgraph (Zhang and Wiemann, 2009) from bioconductor.

1.3 Convergence of Markov chains

Convergence of Markov chains was checked using the Gelman and Brooks method (Brooks and Gelman, 1998). The method is based on running several chains from overdispersion of the starting values, then comparing the variance of the stationary distribution and within-chain variance. The square root of the ratio, termed "potential scale reduction factor", is used as closeness measure. When the variances are far from each other(potential scale reduction factor$\gg$1) then we run our chains longer to improve convergence to the stationary distribution.The variance of the stationary distribution is approximated based on the weighted sum of the within and between chain variance. The average potential scale reduction factors for each of the runs in the manuscript are shown in table 1, which indicates that all chains are converged.

Table 1: Convergence of Markov chains

	Mean	Standard devaition
SN1	1.18	0.15
SN2	1.22	0.12
ERBB	1.32	0.26

Average of potential scale reduction factor of all chains in the population for each model.

2 Results

For the evaluation of the proposed network inference model, we compared the performance of the D-PBTN, DEPN, BDAGL und PBTN approaches on incomplete data, noisy data and KEGG networks. ROC-curves of results are shown in the main manuscript, PR-Curve are shown in supplementary figures S1 to S3, respectively. Supplementary figure S4 shows the distributions of edge weights for inference of the ERBB network with and without prior. To analyze the robustness of network inference with respect to variation of model hyperparameters, we changed the parameters in the model up to ±50% from the values used in reconstruction. The effect of their variation on method performance is shown in supplementary figures S5 to S7. Supplementary figure S8 shows the reference network from STRING used for the ERBB data set.

References

Brooks, P. and Gelman, A. (1998). General Methods for Monitoring Convergence of Iterative Simulations. *J Comput Graph Stat*, **7**, 434–455.

Hua, B. and Tsui, K. (2010). Distributed evolutionary monte carlo for bayesian computing. *Comput Stat Data An*, **54**, 688–697.

Zhang, J. D. and Wiemann, S. (2009). *KEGGgraph: KEGGgraph: A graph approach to KEGG PATHWAY in R and Bioconductor*. R package version 1.2.2.
