# Supplementary figures and images for: Dynamic probabilistic threshold networks to infer signaling pathways from time-course perturbation data
Source: BMC Bioinformatics. 2014 Jul 22;15(1):250. doi: 10.1186/1471-2105-15-250 (PMC4133630; doi:10.1186/1471-2105-15-250)

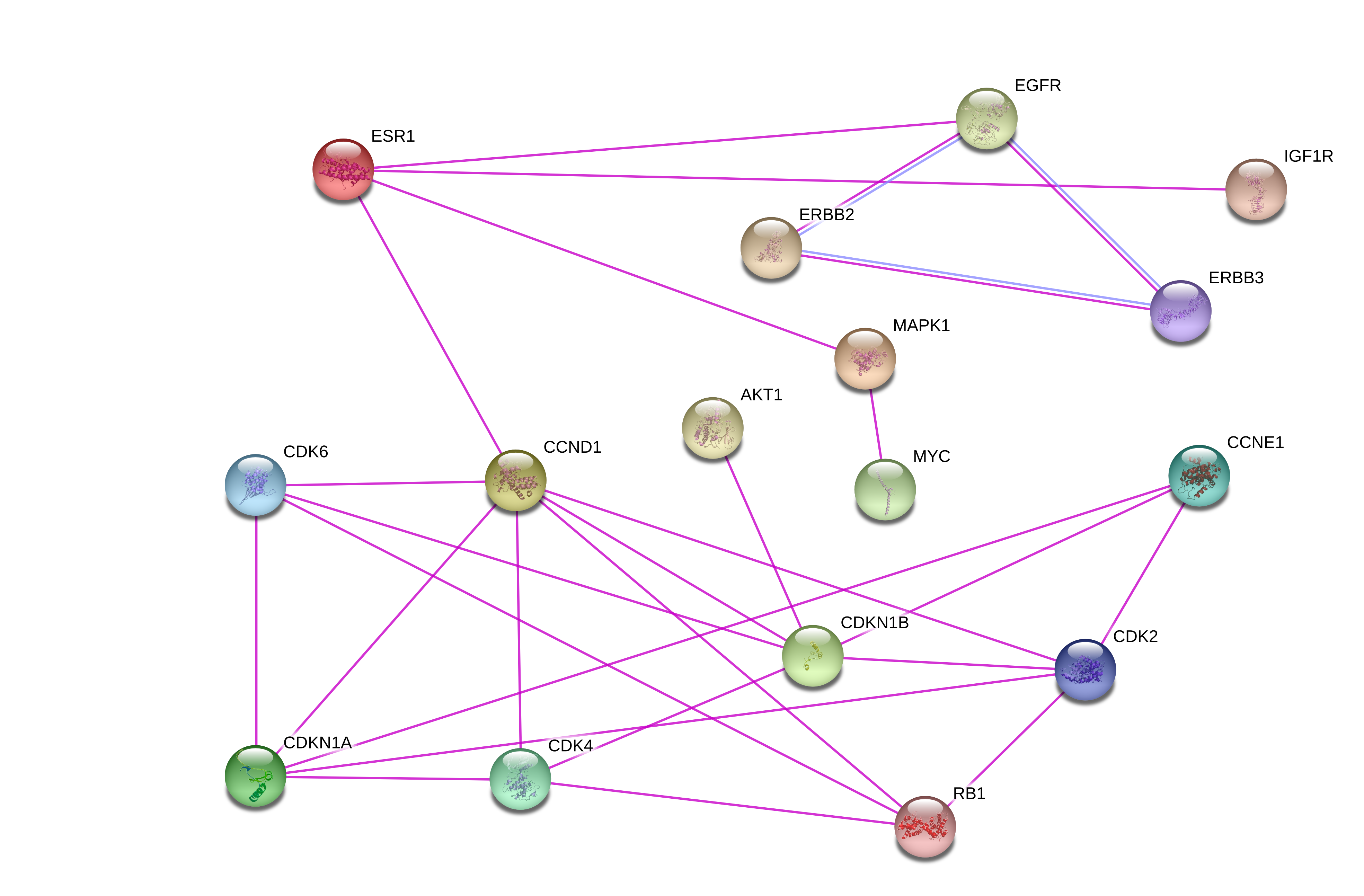

Supplement: Supplementary file 9 — Additional file 9: Supplementary Figure S8. Experimental reference network extracted from STRING for ERBB network inference. (PNG 1 MB) [file 12859_2013_6530_MOESM9_ESM.png]
